# Insulin Signaling Disruption and INF-γ Upregulation Induce Aβ_1–42_ and Hyperphosphorylated-Tau Proteins Synthesis and Cell Death after Paraquat Treatment of Primary Hippocampal Cells

**DOI:** 10.1021/acs.chemrestox.2c00278

**Published:** 2022-11-17

**Authors:** Maria Luisa Abascal, Javier Sanjuan, Paula Moyano, Emma Sola, Andrea Flores, José Manuel Garcia, Jimena Garcia, María Teresa Frejo, Javier del Pino

**Affiliations:** † Department of Pharmacology and Toxicology, Veterinary School, Complutense University of Madrid, 28040 Madrid, Spain; ‡ Department of Pathology, Gregorio Marañon Hospital, 28007 Madrid, Spain

## Abstract

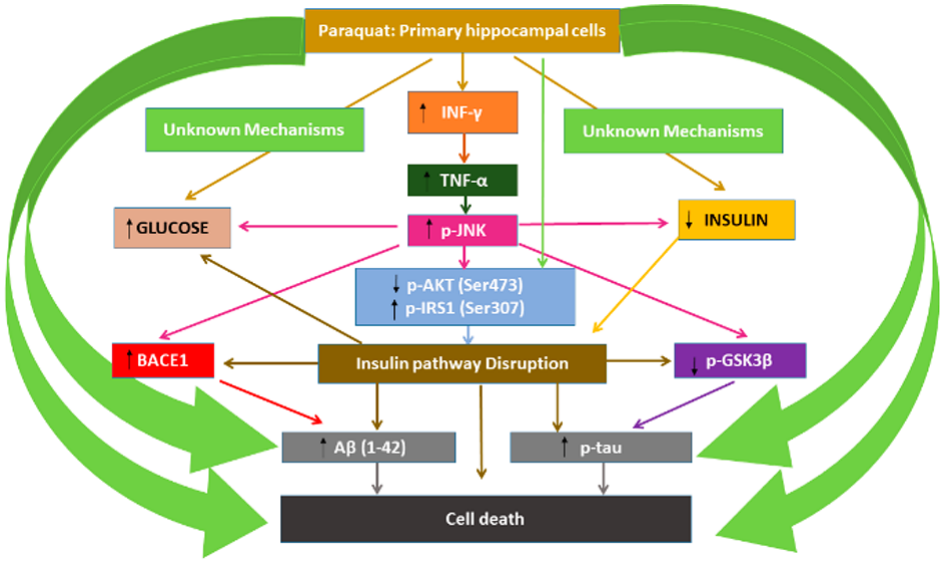

Acute and long-term paraquat (PQ) exposure produces hippocampal neurodegeneration and cognition decline. Although some mechanisms involved in these effects were found, the rest are unknown. PQ treatment, for 1 and 14 days, upregulated interferon-gamma signaling, which reduced insulin levels and downregulated the insulin pathway through phosphorylated-c-Jun N-terminal-kinase upregulation, increasing glucose levels and the production of Aβ_1–42_ and phosphorylated-tau, by beta-site amyloid precursor protein cleaving enzyme 1 (BACE1) overexpression and phosphorylated-GSK3β (p-GSK3β; ser9) level reduction, respectively, which induced primary hippocampal neuronal loss. This novel information on the PQ mechanisms leading to hippocampal neurodegeneration could help reveal the PQ actions that lead to cognition dysfunction.

Paraquat (PQ), a bipyridyl herbicide extensively used, especially in developing countries, produces cognitive decline following acute and repeated exposure, but some of the processes that led to this effect remain unknown.^1,2^ Cognitive function is mediated by the coordinated action of different brain regions, among which the hippocampus is a core center for its development.^3^ Hippocampal neuronal loss, as observed in dementias like Alzheimer’s disease (AD), induces cognition dysfunction.^3^ PQ is redistributed and gathers in the hippocampus,^4^ triggering neuronal damage and loss, following single and long-term exposures, which are associated with the cognitive decline induced by PQ.^1,2,5^ PQ induced hippocampal neurodegeneration, in part, by beta-amyloid (Aβ) and phosphorylated-tau (p-tau) proteins, glutamatergic neurotransmission dysfunction, oxidative stress (OS) generation, BDNF/TrkB/P75^NTR^, and PGE2/EP1–4 signaling pathways disruption, among other actions.^5,6^ However, other mechanisms are involved.

PQ increases interferon-gamma (IFN-γ) blood levels,^7^ and INF-γ knockout in mice reverts the PQ-induced expression of c-Jun-N-terminal-kinase (JNK), activator of transcription-1 (STAT1), and proinflammatory cytokines as tumor-necrosis-factor-α (TNF-α) and the neuronal loss induced in substantia nigra.^8^ Long-term treatment with INF-γ in mice induces hippocampal neurodegeneration and cognitive decline through Janus kinases (JAK)/STAT1 pathway,^9^ which is induced by TNF-α upregulation.^10^ Thus, PQ could mediate hippocampal neurodegeneration through IFN-γ upregulation.

Additionally, PQ decreases and increases blood insulin and glucose levels, respectively,^11^ and decreases insulin signaling activation in 3T3-L1 adipocytes cells through activity repression of its downstream targets phosphatidylinositol-3-kinase (PI3K) and protein-kinase-beta (AKT) by reduction of their phosphorylation, without any action on insulin receptors (IR) and insulin receptor substrates (IRS). These initiate the pathway activation after autophosphorylation of different tyrosine residues.^12^ Insulin signaling pathway disruption, reported in AD and other neurodegenerative diseases, was associated with neuronal loss and cognitive disorders.^10^ Besides, IFN-γ was reported to decrease insulin levels^13^ and insulin pathway activation via JAK/STAT1.^14^ TNF-α was described to induce insulin resistance through increased phosphorylation of IRS1 serine residues, mediated by JAK/STAT1 pathway upregulation.^10^ Therefore, PQ could mediate the insulin signaling disruption by IFN-γ signaling upregulation through JAK pathway upregulation, which could induce the hippocampal neuronal loss described.

Otherwise, IFN-γ and insulin signaling pathways disruption increase Aβ and p-tau proteins.^10,15,16^ Phosphorylated-tau and Aβ proteins accumulation could be due to an increment in their production through increased GSK3β activity that phosphorylated tau proteins and through amyloid precursor protein (βAPP) processing to amyloid peptides by increasing the β-secretase enzyme β-site amyloid precursor protein cleaving enzyme 1 (BACE1) expression or through a reduction of their clearance.^10,15^ IFN-γ and insulin signaling pathway disruption induce GSK3β activity^10,16^ and βAPP and BACE1 overexpression,^10,16^ leading to Aβ and p-tau proteins accumulation. PQ increases BACE1 levels and γ-secretase and GSK3β activities,^2,17,18^ suggesting it could increase Aβ and p-tau production. Besides, PQ was shown to reduce Aβ and p-tau clearance, in part, through HSP70 and TFEB downregulation and proteasome P20S activity inhibition.^19^ Thus, PQ could induce the Aβ and p-tau accumulation through the induction of its production and a reduction of its clearance, mediated by IFN-γ and insulin signaling pathways disruption.

Accordingly, we hypothesized that PQ could disrupt insulin signaling pathways through IFN-γ signaling upregulation, which increases Aβ and p-tau proteins production and reduces their degradation, finally triggering hippocampal neuronal death. To test this hypothesis, we treated unsilenced or TNF-α, βAPP, JNK, IFN-γ, BACE1, GSK3β, and tau siRNA primary hippocampal neurons with 0.1 μM to 40 μM PQ alone or together with 200 nM insulin (Sigma, Madrid, Spain), for 1 and 14 days.

The protocol by del Pino et al.^5^ was followed for cultures of primary hippocampal neurons from rat fetuses, to prove our hypothesis. In the culture, neurons and astrocytes were identified and quantified using MAP2 and GFAP antibodies, respectively, with neurons being predominant (91%) in the cultures. PQ concentrations were chosen because they were previously reported to cause Aβ and p-tau proteins aggregation and apoptosis in primary culture hippocampal cells following 1 and 14 days treatment as well as cell death following intrahippocampal injection in rats.^5,6^ The 10 μM PQ concentration was chosen to test the role of the mechanisms proposed because it was the lowest concentration that produces neuronal death and increases Aβ and p-tau peptides after a single treatment.^5,6^

The viability of primary hippocampal neurons was studied, after 1 or 14 days of PQ treatment, using the MTT test.^5^ Induction of apoptotic death was determined with Caspase-Glo 3/7 luminescence assay kit (Promega, Madrid, Spain), following the manufacturer’s directions. Following the producer’s instructions, glucose levels were analyzed with a commercial kit (Abcam, Madrid Spain). Commercial ELISA kits (Invitrogen, Madrid Spain) were performed to analyze insulin and tau and Aβ proteins’ concentrations, according to the manufacturer’s protocol.

Validated primers (SA Biosciences) for mRNAs encoding ACTB (PPR06570C), tau (PPR42757D), INF-γ (PPR45050C), βAPP (PPR06788A), TNF-α (PPR06411F), insulin (PPR42359A), GSK3β (PPR44848A), BACE1 (PPR50333A), and JNK (PPR43333A) were employed, following the protocol of del Pino et al.,^5^ for gene expression analysis. The cycle threshold (Ct) method^20^ was followed to analyze QPCR data. INF-γ, TNF-α, p-IRS1 (panTyr), p-IRS1 (Ser307), p-AKT(Ser473), BACE1, GSK3β (Ser9), and p-JNK(Ser63) proteins expressions were analyzed with commercial ELISA kits (MBS031457, MBS697379, MBS1607535, MBS1605652, MBS9501391, MBS1600225, MBS9511030, and MBS2605744, respectively, MyBioSource, CA, USA), following the producer’s guideline. siRNA (Qiagen, Barcelona, Spain) homologous to rat βAPP (SI01488767), INF-γ (SI01524250), TNF-α (SI02046583), tau (SI02876027), JNK (SI03083185), GSK3β (SI01519406), and BACE1 (SI03082247) genes were used to transfect cells.^5,6^ siRNA treatment efficiency was tested by analysis of gene expression (Figure S1). PQ treatment of cells transfected with scrambled siRNA showed no significant difference with PQ treatment of wild-type cells (data not shown). Reported results represent all (at least three experiments) replicates (in triplicate) performed for each experimental condition (*n* = 9). Results are presented as means ± standard error of the mean (SEM). ANOVA analyses (one-way for concentration–response analysis, and two-way for gene silencing/treatment), followed by Tukey posthoc test, were performed to identify statistically significant differences between treatments (*p* ≤ 0.05), using the GraphPad software (GraphPad Software, Inc, San Diego, CA, USA).

PQ increased IFN-γ, TNF-α, p-JNK (Ser63), and p-IRS1 (Ser307) protein levels (Figure 1), but reduced insulin and p-IRS1 (panTyr) protein levels (Figure 1) and insulin gene expression (data not shown), following 1 (starting at 10 μM) and 14 days of treatment (starting at 1 μM). The PQ effect on TNF-α and p-JNK (Ser63) levels was partially abolished by IFN-γ or TNF-α silencing (Figure 1), respectively, showing that PQ upregulates IFN-γ, which increases TNF-α, and finally this increases p-JNK (Ser63) levels. Additionally, the PQ effect on insulin and p-IRS1 (Ser307) and p-IRS1 (panTyr) protein levels was partially abolished after PQ treatment of JNK silenced cells, pointing out that PQ mediates insulin signaling disruption, in part, through IFN-γ signaling upregulation.

**Figure 1:**
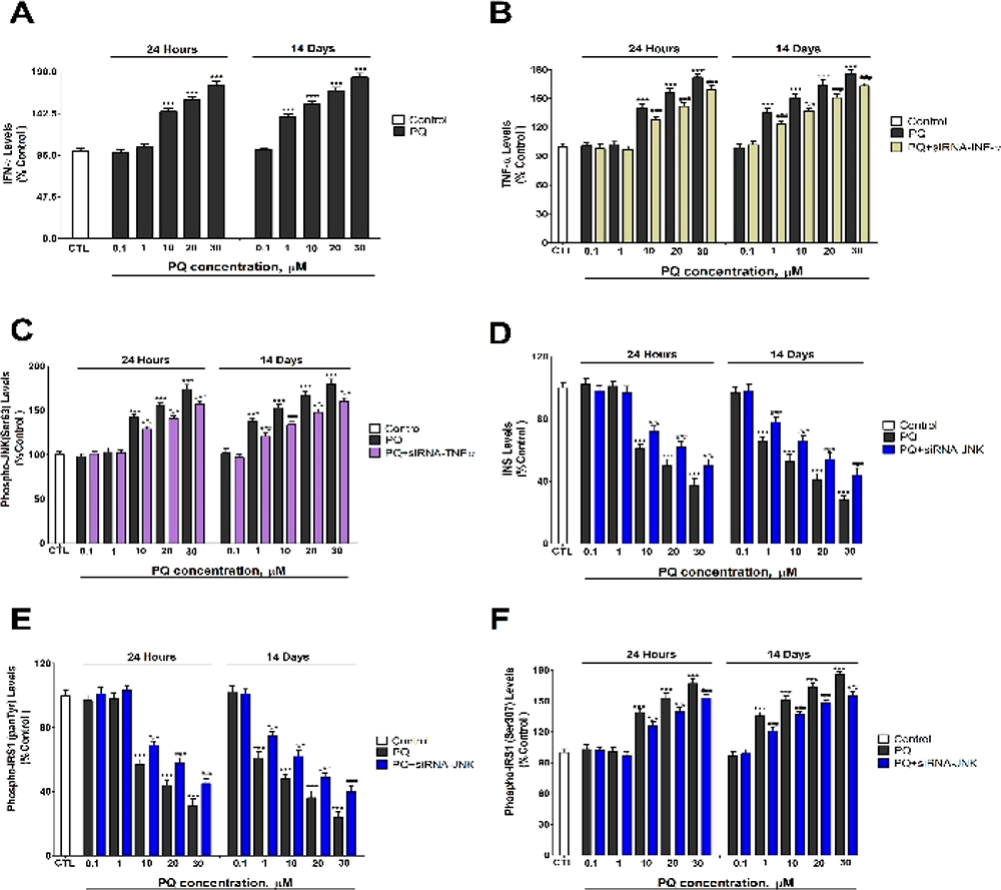
IFN-γ (A), TNF-α (B), p-JNK (C), insulin (D), p-IRS1 (panTyr) (E), and p-IRS1 (Ser307) (F) levels analysis. Each bar represents mean ± SEM of all replicates of experiments from three individual experimental conditions. ***p ≤ 0.001, significantly different from controls. ^###^*p* ≤ 0.001 significantly different from PQ treatment.

PQ was shown to increase IFN-γ blood levels,^7^ and revert the expression of JNK, and TNF-α in substantia nigra through INF-γ silencing,^8^ supporting our findings. Insulin was shown to be expressed in the hippocampus,^21^ and, although the PQ effect on brain insulin levels was not studied, PQ exposure decreases insulin blood levels,^12^ supporting our results. However, PQ was shown not to alter IRS-1 tyrosine phosphorylation after PQ or PQ+insulin treatment of primary rat hepatocytes.^22^ This contradiction could be due to the different times of exposure, concentrations, or model of cells used. IFN-γ decreases insulin levels^13^ and insulin signaling pathway activation through JAK/STAT1 pathway upregulation,^14^ which increases IRS1 phosphorylation of serine residues,^10^ supporting the results observed. The Wnt pathway regulates the insulin pathway,^23^ and PQ disrupts the Wnt signaling pathway.^24^ Thus, the PQ Wnt pathway alteration could also mediate the insulin pathway disruption.

PQ also decreased p-AKT (Ser473) (Figure 2A,B) and p-GSK3β protein levels (Figure 3A,B) and increased glucose levels (Figure 2C,D), following 1 (starting at 10 μM) and 14 days of treatment (starting at 1 μM). Besides, PQ increased BACE1, Aβ_1–42_, and p-tau protein levels (Figure 3). PQ decreases the local insulin synthesis and induces slight insulin resistance, leading to an increase in glucose levels, since insulin induced glucose utilization by the cells. PQ decreases p-AKT (Ser473) in SH-SY5Y cells,^25^ which supports our findings, but was shown also to increase it in rat primary hepatocytes cells.^22^ In the cell model used, concentration or exposure time may be responsible for these differences. PQ also increases blood glucose levels because of hypoinsulinemia,^11^ supporting our results. Besides, PQ was shown to increase BACE1 protein levels in the mouse cortex,^2^ supporting our findings, but to decrease p-GSK3β (Tyr216) protein levels in rat hippocampus,^17^ which indicates GSK3β activation and increased activity. These differences could be due to differences in the time of exposure and doses used. Finally, PQ increases, in a concentration dependent-way, the Aβ_1–42_ and p-tau levels in the rat hippocampus and primary hippocampal cells, confirming our results.^1,2,6,19^

**Figure 2:**
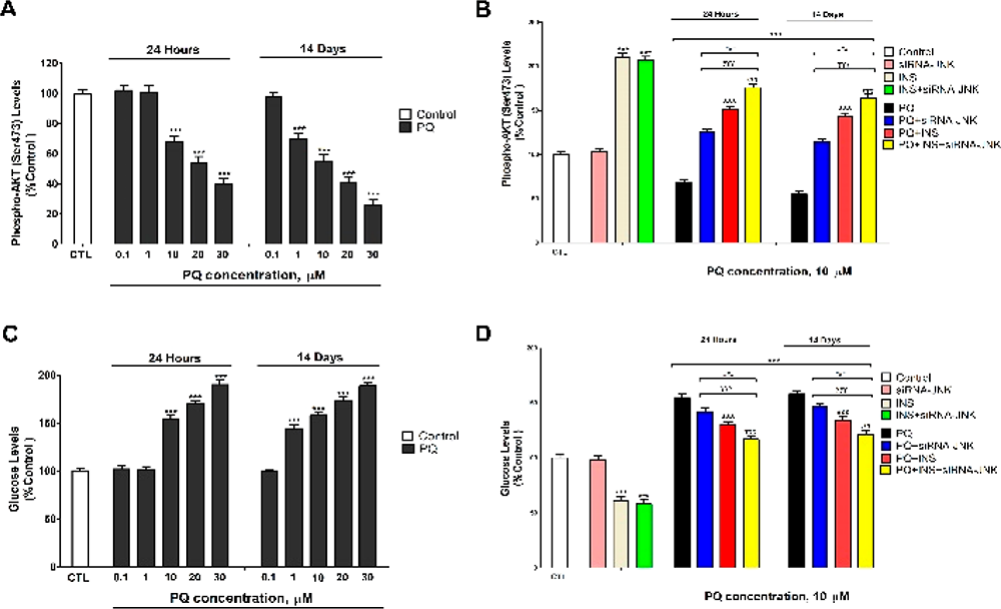
Phosphorylated-AKT (Ser473) (A, B) and glucose (C, D) levels analysis. Each bar represents mean ± SEM of all replicates of experiments from three individual experimental conditions. The values obtained were normalized by total protein concentrations. ***p ≤ 0.001 correlated to control. ^###^*p* ≤ 0.001 correlated to PQ treatment. ^&&&^*p* ≤ 0.001 correlated to PQ treatment of JNK silenced cells. ^τττ^*p* ≤ 0.001 compared to PQ+insulin treatment. ^γγγ^*p* ≤ 0.001 compared to insulin treatment.

**Figure 3:**
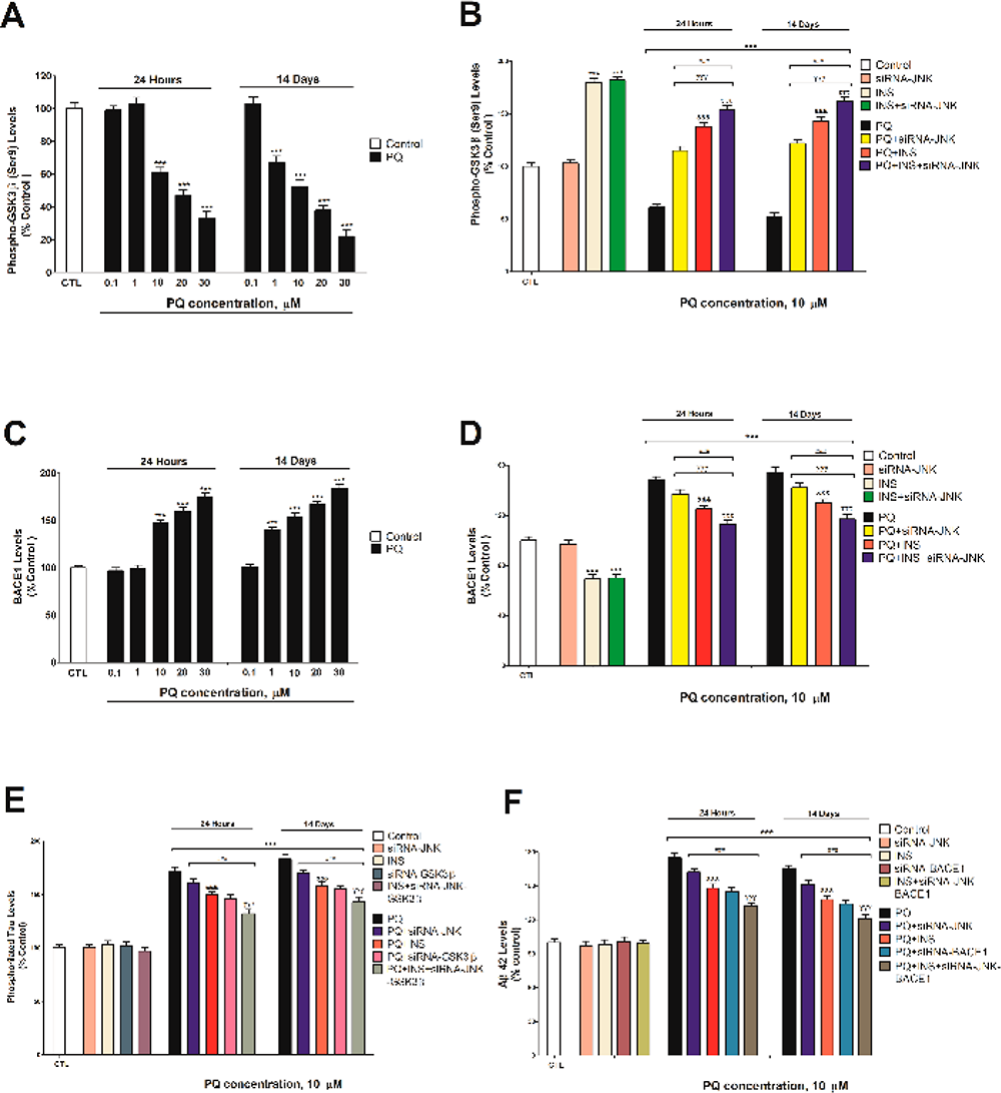
Phosphorylated-GSK3β (Ser9) (A, B), BACE1 (C, D), p-tau (E), and Aβ_1–42_ (F) levels analysis. Each bar represents mean ± SEM of all replicates of experiments from three individual experi-mental conditions. The values obtained were normalized by total protein concentrations. ***p ≤ 0.001 correlated to control. ^###^*p* ≤ 0.001 correlated to PQ treatment. ^&&&^*p* ≤ 0.001 correlated to PQ treatment of JNK silenced cells. ^τττ^*p* ≤ 0.001 compared to PQ+insulin treatment. ^γγγ^*p* ≤ 0.001 compared to PQ treatment of BACE1 silenced cells.

PQ treatment of JNK silenced cells or of unsilenced cells with insulin reversed partially the effects on glucose, p-AKT (Ser473), p-GSK3β, BACE1, Aβ_1–42_, and p-tau proteins levels observed after the PQ single treatment of wildtype cells (Figure 2 and 3). PQ cotreatment of JNK silenced cells with insulin induced the higher reversion observed on the targets commented, but it was incomplete (Figure 2 and 3). Additionally, Aβ_1–42_ and p-tau increased levels were reversed also after PQ treatment of the BACE1 silenced cells in the case of Aβ_1–42_ or after PQ treatment of the GSK3β silenced cells in the case of p-tau (Figure 3). PQ cotreatment of JNK and BACE silenced cells with insulin or cotreatment of JNK and GSK3β silenced cells with insulin induced the higher reversion observed in the increment of Aβ_1–42_ and p-tau levels, but was still incomplete (Figure 3). These results point out that the INF-γ pathway downregulates the insulin signaling pathway, which increases the production of Aβ_1–42_, through BACE1 overexpression, and p-tau proteins, through GSK3β activation.

IFN-γ and insulin signaling pathways upregulation and downregulation, respectively, induce GSK3β activity,^10,16^ BACE1 overexpression,^10,16^ and increase Aβ and p-tau proteins levels,^10,15,16^ supporting these findings. However, other mechanisms seem to be involved. In this sense, insulin signaling pathway disruption decreases insulin degrading enzyme expression,^10^ which participates in Aβ proteins clearance,^10^ so this mechanism could also contribute. Besides, PQ was shown to reduce Aβ and p-tau clearance, in part, through HSP70 and TFEB downregulation and proteasome P20S activity inhibition.^19^ Insulin regulates HSP70 expression,^25^ proteasome P20 activity,^26^ and mammalian target of rapamycin (mTOR) activity,^27^ which downregulate TFEB.^19^ Therefore, PQ could induce the Aβ and p-tau accumulation through its production and a reduction of its clearance through IFN-γ and insulin signaling pathways disruption mediated by the commented mechanisms.

Finally, PQ (10 μM), following 1 and 14 days of treatment, decreased cell viability (52.1% and 41.6%, respectively; Figure 4A) and induced apoptotic cell death (172.2% and 191.9%, respectively; Figure 4B), as previously reported.^5,6,19^ PQ treatment of primary hippocampal neurons single silenced against βAPP, JNK, or tau or cotreatment with insulin of wildtype cells reverse partially the hippocampal neuronal loss produced (Figure 4). PQ cotreatment of simultaneous βAPP, JNK, and tau silenced cells with insulin produced the higher reversion of the cell death observed after PQ treatment alone, but was not complete (Figure 4). PQ induces primary hippocampal neuron cell death through an increase of Aβ and p-tau levels.^6^ PQ induces substantia nigra neuronal loss through INF-γ silencing.^8^ INF-γ long-term treatment induces hippocampal neurodegeneration and cognitive decline in mice,^9^ and PQ treatment of IFN-γ knockout mice reverted the substantia nigra neuronal loss induced after PQ treatment alone.^8^ Insulin signaling pathway disruption induces hippocampal neuronal loss and produces cognitive disorders, and insulin treatment reverts these effects.^10,28,29^ All these previous studies support our results. PQ induces OS,^1,2^ which could induce neuroinflammation,^7,8^ Aβ proteins accumulation,^2^ and cell death,^6^ so PQ could mediate the effect observed through OS generation. Finally, our results point out that additional mechanisms may participate in the hippocampal neuronal loss produced. The Wnt signaling pathway maintains cognitive function and cell viability.^23,30^ PQ disrupts the Wnt pathway;^23^ therefore, this mechanism together with those previously described could contribute to the neuronal loss observed and the cognitive decline described after PQ treatment.

**Figure 4:**
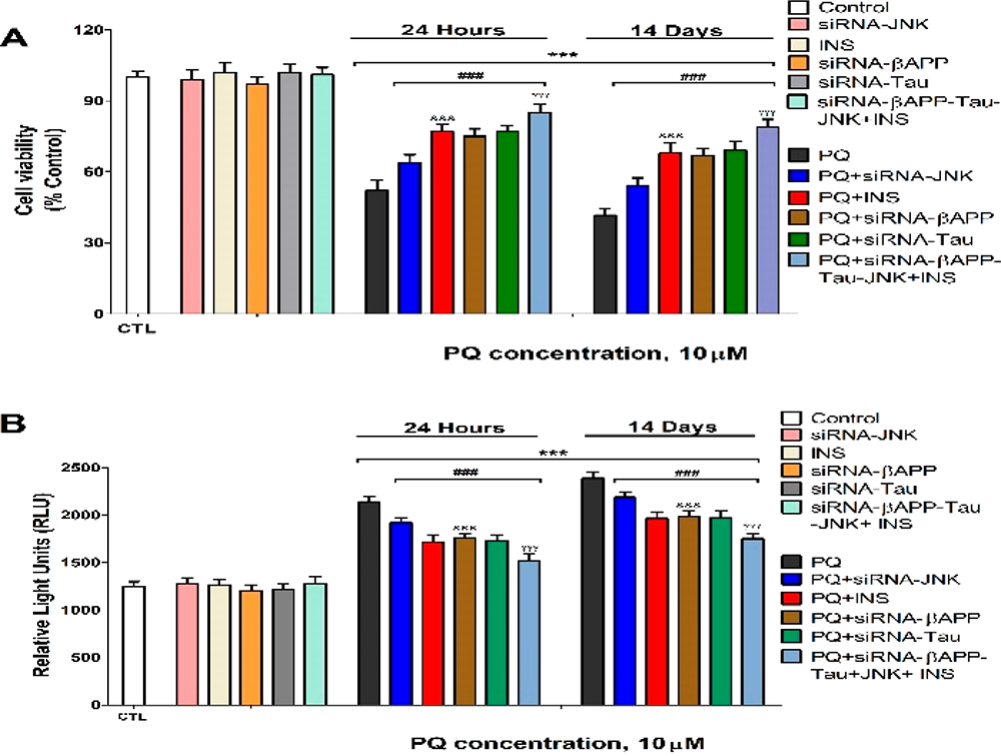
Cell viability (A) and caspases 3/7 activity (B) analyses. Each bar represents mean ± SEM of three separate experiments from cells of different cultures, each one performed in triplicate. The values obtained were normalized by total protein concentrations. ***p ≤ 0.001 correlated to control. ^###^*p* ≤ 0.001 correlated to PQ exposure. ^&&&^*p* ≤ 0.001 correlated to PQ+insulin treatment. ^γγγ^*p* ≤ 0.001 compared to tau siRNA cells exposed to PQ.

Accordingly, PQ (1 and 14 days of treatment) upregulates the INF-γ signaling pathway that produces a reduction of insulin levels and insulin signaling pathway downregulation through JNK, which induces Aβ_1–42_ and tau peptides production and hippocampal neuronal loss. Further studies are required to determine the unknown mechanism through which PQ also disrupts the insulin signaling pathway, mediates the Aβ_1–42_ and tau peptides production, and induces hippocampal neuronal loss. The PQ effect on insulin and INF-γ produced peripherally could contribute to the local effect observed, and it is necessary to perform *in vivo* studies to corroborate that these mechanisms are produced and the local and peripheral contribution to the effect observed and the induction of cognitive decline. Our results provide new information on PQ toxic mechanisms that may lead to hippocampal neurodegeneration, which could clarify the PQ toxic action that triggers cognitive dysfunction and provide additional tools to prevent and manage these processes.

## Supporting Information Available

Figure S1: Transfection efficiency for siRNA (PDF)
