# Supplementary material for: Insulin Signaling Disruption and INF-γ Upregulation Induce Aβ1–42 and Hyperphosphorylated-Tau Proteins Synthesis and Cell Death after Paraquat Treatment of Primary Hippocampal Cells
Source: Chem Res Toxicol. 2022 Nov 17;35(12):2214–8. doi: 10.1021/acs.chemrestox.2c00278 (PMC9768806; doi:10.1021/acs.chemrestox.2c00278)
Supplement: Supplementary file 1 — tx2c00278_si_001.pdf [file tx2c00278_si_001.pdf]

Supporting Information

Insulin signaling disruption and INF-γ upregulation induce Aβ$_{1-42}$ and hyperphosphorylated-Tau proteins synthesis and cell death after paraquat treatment of primary hippocampal cells

Maria Luisa Abascal[2], Javier Sanjuan[1], Paula Moyano[*1], Emma Sola[*1], Andrea Flores,[1] José Manuel Garcia[1], Jimena Garcia[1], María Teresa Frejo[1], and Javier del Pino[*1]

[1] Department of Pharmacology and Toxicology, Veterinary School, Complutense University of Madrid, 28040, Madrid, Spain.
[2]Department of Pathology, Gregorio Marañon Hospital, 28007, Madrid, Spain.

Figure S1.

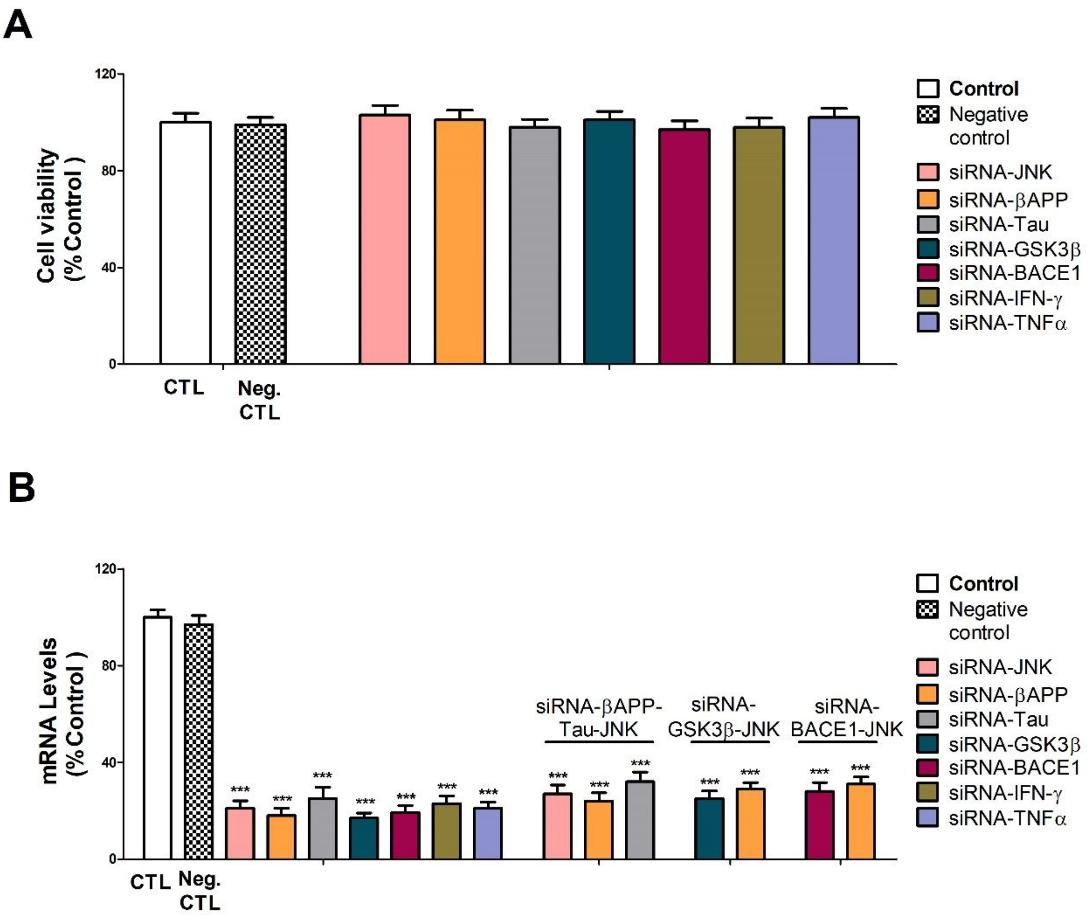

Figure S1. A) shows cell viability of INF-γ, TNF-α, JNK, GSK3β, BACE1, βAPP and Tau knockdown primary hippocampal cells evaluated by MTT test. B) shows silencing efficiency evaluated by gene expression analysis. Control: Primary hippocampal cells transfected without siRNA. Negative (Neg.) control: cells transfected with scrambled siRNA. INF-γ-siRNA: transfected with siRNA against INF-γ. TNF-α-siRNA: transfected with siRNA against TNF-α. JNK-siRNA: transfected with siRNA against JNK. GSK3β-siRNA: transfected with siRNA against GSK3β. BACE1-siRNA: transfected with siRNA against BACE1. βAPP-siRNA: transfected with siRNA against βAPP. Tau-siRNA: transfected with siRNA against Tau. βAPP+Tau+JNK-siRNA: transfected with siRNA against JNK, βAPP and Tau. GSK3β+JNK-siRNA: transfected with siRNA against JNK and GSK3β. BACE1+JNK-siRNA: transfected with siRNA against JNK and BACE1. Values are given as mean ± SEM. $^{***}p \leq 0.001$ compared to control.
